# Reduced glutathione enhances adipose tissue‐derived mesenchymal stem cell engraftment efficiency for liver fibrosis by targeting TGFβ1/SMAD3/NOX4 pathway

**DOI:** 10.1002/btm2.10735

**Published:** 2024-12-10

**Authors:** Shaoxiong Yu, Yingchao Wang, Yingjun Shi, Saihua Yu, Bixing Zhao, Naishun Liao, Xiaolong Liu

**Affiliations:** ^1^ The United Innovation of Mengchao Hepatobiliary Technology Key Laboratory of Fujian Province Mengchao Hepatobiliary Hospital of Fujian Medical University Fuzhou China; ^2^ Mengchao Med‐X Center Fuzhou University Fuzhou China; ^3^ The Liver Center of Fujian Province Fujian Medical University Fuzhou China

**Keywords:** adipose tissue‐derived mesenchymal stem cells, cell engraftment efficiency, reactive oxide species, reduced glutathione, TGFβ1/SMAD3/NOX4 signaling

## Abstract

Reduced glutathione (GSH) could reduce oxidative stress to improve adipose tissue‐derived mesenchymal stem cell (ADSC) engraftment efficiency in vivo. However, the underlying mechanisms remain unclear. Our goal is to investigate whether GSH enhances ADSC engraftment through targeting the TGFβ/SMAD3/NOX4 pathway. Liver fibrotic male mice were administrated GSH, setanaxib (STX), and SIS3 during ADSC transplantation. ADSC engraftment efficiency and reactive oxygen species (ROS) level were detected both in vivo and ex vivo. Biochemical analysis was used to analyze the content of superoxide and nicotinamide adenine dinucleotide phosphate oxidases (NOXs) in liver tissues. Immunohistochemistry and western blotting were used to examine the protein level of NOX1, NOX2, NOX4, transforming growth factor‐β1 (TGFβ1), SMAD3, and p‐SMAD3 in liver tissues. Additionally, the therapeutic efficacy of the ADSC transplantation was further investigated. We found that GSH significantly improved ADSC engraftment efficiency, which was closely related to the reduced ROS generation in liver tissues. However, the enhanced cell engraftment was abolished after the combined treatment with STX or SIS3. GSH could effectively reduce superoxide and NOXs content, and selectively inhibit NOX4 expression in liver tissues. The co‐localization results showed that GSH could reduce NOX4 expressed in activated hepatic stellate cells. Mechanistically, GSH down‐regulated TGFβ/SMAD3 signaling. More importantly, GSH enhanced the therapeutic efficacy of ADSC therapy in liver fibrotic mice. Taken together, GSH could improve the engraftment efficiency of ADSCs in liver fibrosis by targeting TGFβ1/SMAD3/NOX4 signaling pathway, which provides a new theoretical basis for GSH enhancing ADSC engraftment efficiency in liver diseases.

**Translational Impact Statement**

Our study demonstrates that reduced glutathione (GSH) significantly enhances the engraftment efficiency of adipose‐derived stem cell (ADSC) in liver fibrosis by targeting the TGFβ1/SMAD3/NOX4 signaling pathway. This finding highlights GSH's potential to improve ADSC‐based therapies for liver diseases, providing a novel approach to reduce oxidative stress and promote cell engraftment. These results pave the way for improved clinical outcomes in liver fibrosis treatment, emphasizing the therapeutic value of GSH in enhancing stem cell efficacy and offering a promising strategy for regenerative medicine.

## 1 INTRODUCTION

Adipose tissue‐derived mesenchymal stem cell (ADSC) therapy provides a promising strategy for various liver diseases, ranging from acute to chronic liver diseases.^1^, ^2^, ^3^ Particularly, pre‐clinical studies have shown the potential effectiveness of ADSC therapy in alleviating end‐stage liver diseases.^4^, ^5^ However, the low hepatic engraftment efficiency of ADSCs hampers further clinical translation of ADSC transplantation for liver diseases.^6^, ^7^ There are many methods, including gene modification and nanomaterials,^8^, ^9^, ^10^ have been found to improve the stem cell engraftment efficiency. However, these strategies have some disadvantages that are unfavorable for clinical translation of ADSC therapy.^11^, ^12^, ^13^ For example, gene modification requires the insertion of an exogenous virus vector that will bring unpredictable risk for the whole system in vivo^14^, ^15^; the long‐term toxicity, biocompatibility and the quality control of nanomaterials are still difficult to fully verify in further clinical practice during ADSC therapy. Hence, there is an urgent need to find potential clinically available strategies that can effectively enhance ADSC engraftment efficiency for liver diseases.^16^, ^17^

Previous studies have indicated that ADSC biological characteristics, including stemness, proliferation, and migration, are prone to being injured by oxidative stress both in vitro and in vivo.^18^, ^19^ Taking into consideration of this limitation, in vitro antioxidant preconditioning, including melatonin and reduced glutathione (GSH), has been used to enhance ADSC engraftment efficiency by reducing reactive oxygen species (ROS)‐induced injury.^20^ Furthermore, considering that excessive ROS generation of in vivo can also be reduced by antioxidant treatment, it can be inferred that in vivo reducing hepatic ROS level also benefits to improve ADSC engraftment efficiency in liver diseases. Indeed, we previously found that GSH, a well‐known hepatoprotective antioxidant that has been extensively utilized in clinical practice, effectively improves ADSC engraftment efficiency in liver fibrosis.^21^ A comprehensive understanding of the underlying mechanisms should be further explored to confirm the clinical application potential of GSH for enhancing ADSC therapy in liver diseases.

Nicotinamide adenine dinucleotide phosphate (NADPH) oxidases (NOXs) are the major sources of ROS production.^22^, ^23^, ^24^ NOX1, NOX2 and NOX4 are the main homologs of NOXs found in the liver.^25^, ^26^ In particular, NOX4 plays a significant role in ROS production, and is highly expressed in hepatocytes, liver sinusoidal endothelial cells, hepatic stellate cells (HSC), and myofibroblasts during the progression of liver fibrosis.^27^, ^28^, ^29^ Although the complete upstream signaling pathways of NOXs remain unknown, TGFβ1/SMAD signaling pathway, a well‐known regulator in the progression of liver diseases,^30^, ^31^ has been shown to regulate NOX4/ROS pathway in a SMAD3‐dependent manner.^32^ We therefore hypothesized that the inhibition of TGFβ1/SMAD3/NOX4 pathway could be a potential mechanism by which GSH enhances ADSC engraftment efficiency in liver diseases. In this study, we aimed to elucidate whether GSH could enhance ADSC engraftment efficiency by inhibiting the TGFβ1/SMAD3/NOX4 pathway using a tetrachloromethane‐injured liver fibrotic mice model.

## 2 RESULTS

### 2.1 GSH improves ADSC engraftment efficiency in liver fibrosis

**FIGURE 1:**
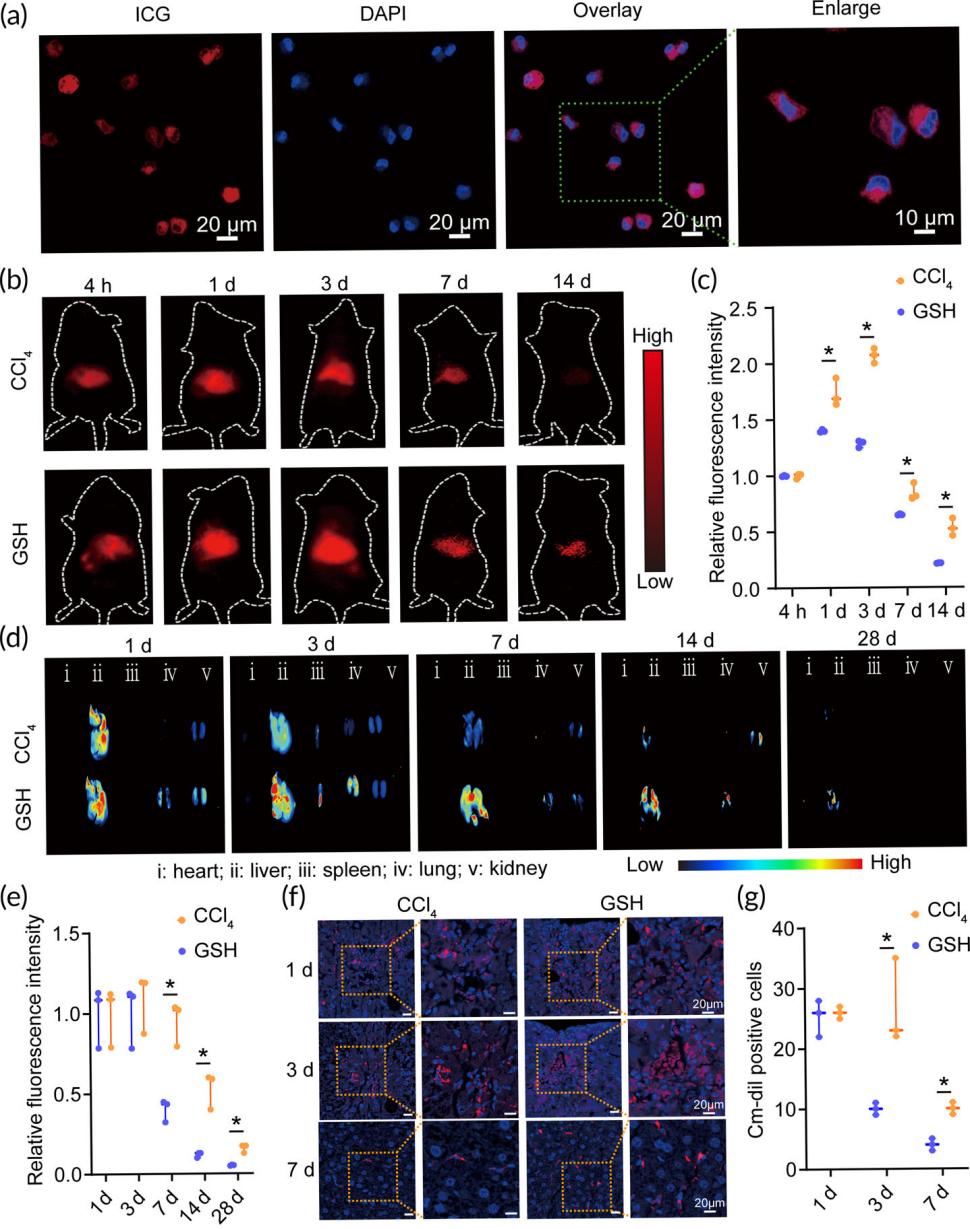
Glutathione (GSH) enhances adipose tissue‐derived mesenchymal stem cell (ADSC) engraftment efficiency in vivo. (a) Representative confocal laser scanning microscopy (CLSM) images of ADSCs after indocyanine green (ICG) labeling. Scale bars: 20 and 10 μm. (b) In vivo near‐infrared (NIR) imaging after ADSC transplantation for 14 days. (c) Relative fluorescence intensity of ADSCs after ADSC transplantation for 14 days. (d) Ex vivo imaging of ADSCs in major organs, including the heart (i), liver (ii), spleen (iii), lung (iv), and kidney (v), after cell transplantation for 28 days. (e) Relative fluorescence intensity of ADSCs in liver tissues after ADSC transplantation for 28 days. (f) CLSM imaging of liver sections after ADSC transplantation for 7 days. Scale bars = 20 μm. (g) The Cm‐dil‐positive ADSCs after cell transplantation for 1, 3, and 7 days, respectively. CCl_4_, carbon tetrachloride. *, indicating *p* < 0.05.

To evaluate ADSC engraftment efficiency in vivo, cell labeling was performed using the food and drug administration (FDA)‐approved infrared fluorescent dye, indocyanine green (ICG). Confocal laser scanning microscopy (CLSM) revealed successful labeling of ADSCs with ICG after 24‐h incubation (Figure 1a). Following a 14‐day period of cell tracking, we observed a notable increase in the ICG signal (Figure 1b) and the relative fluorescence intensity (Figure 1c) in the GSH group compared to the carbon tetrachloride (CCl_4_) treatment (*n* = 3). To validate this result, the ADSC engraftment was also evaluated by Cm‐dil labeling as our previous description.^20^ As predicted, the ex vivo fluorescence imaging showed an enhanced Cm‐dil signal in liver tissues following GSH treatment for 28 days (Figure 1d,e). CLSM imaging further confirmed the increased number of Cm‐dil‐positive ADSCs in liver tissue sections (Figure 1f,g). Taken together, these results suggested that GSH treatment enhances ADSC engraftment efficiency in liver fibrosis.

### 2.2 GSH improves ADSC engraftment efficiency by reducing oxidative stress

**FIGURE 2:**
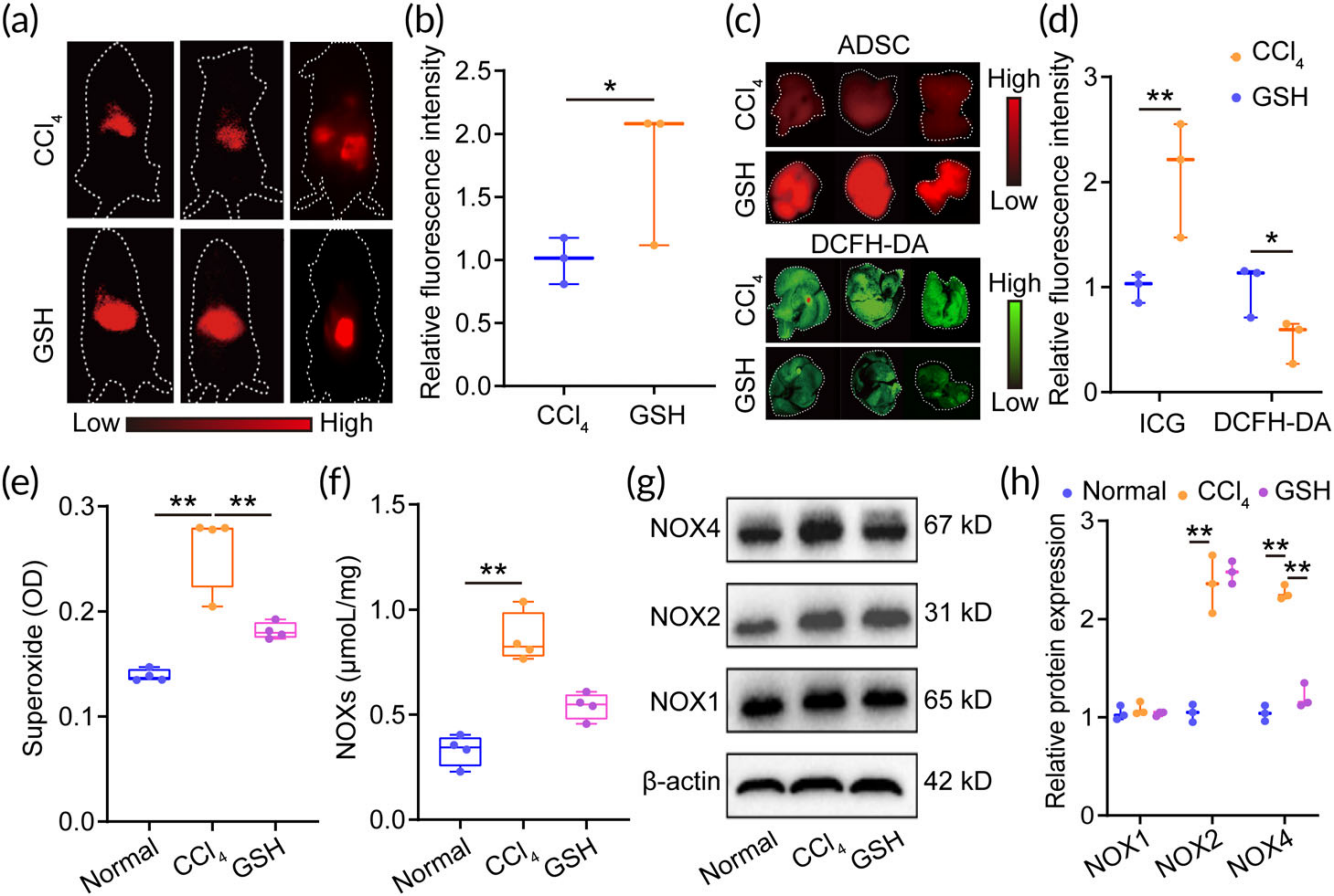
Glutathione (GSH) enhances adipose tissue‐derived mesenchymal stem cell (ADSC) engraftment efficiency by inhibiting oxidative stress and NOX4 expression. (a) In vivo NIR imaging after ADSC transplantation for 3 days. (b) Relative fluorescence intensity of indocyanine green (ICG) signal in vivo. (c) Ex vivo NIR imaging and ROS content in liver tissues. (d) Relative fluorescence intensity of ICG signal and reactive oxygen species in liver tissues. (e) Quantification of superoxide level in liver tissues after GSH treatment. (f) Nicotinamide adenine dinucleotide phosphate oxidases (NOXs) content in liver tissues after GSH treatment. (g) Western blotting of NOX4, NOX2, and NOX1 in liver tissues after GSH treatment. (h) Quantification of NOX4, NOX2, and NOX1 expression in liver tissues. CCl_4_, carbon tetrachloride; DCFH‐DA, 2′,7′‐dichlorofluorescein diacetate; OD, optical density. *, indicating *p* < 0.05; **, indicating *p* < 0.01.

Considering that liver fibrosis is characterized by excessive ROS generation,^33^ which hinders the engraftment of ADSCs in vivo, we next investigated whether the improved efficiency of cell engraftment is correlated with a reduction in ROS levels in vivo. After confirming the enhanced ICG signal (Figure 2a) and the relative fluorescence intensity (Figure 2b) of ADSCs in vivo following GSH treatment for 3 days, we simultaneously analyzed the ICG signal and ROS content in ex vivo liver tissues (*n* = 3). Interestingly, we found the enhanced ICG signal was accompanied by low ROS content (2′,7′‐dichlorofluorescein diacetate [DCFH‐DA] signal) in the GSH group (Figure 2c,d), which means a negative correlation between the ICG signal and ROS content. To further corroborate the antioxidant capability of GSH, we also examined the levels of superoxide in liver tissues. As shown in Figure 2e, GSH exhibited a significant reduction of superoxide levels in fibrotic liver tissues compared to the CCl_4_ treatment, indicating its strong antioxidant properties. These findings suggest that GSH can enhance the efficiency of ADSC engraftment partly by reducing ROS generation and superoxide content in liver tissues.

### 2.3 GSH improves ADSC engraftment efficiency by reducing NOX4 expression

**FIGURE 3:**
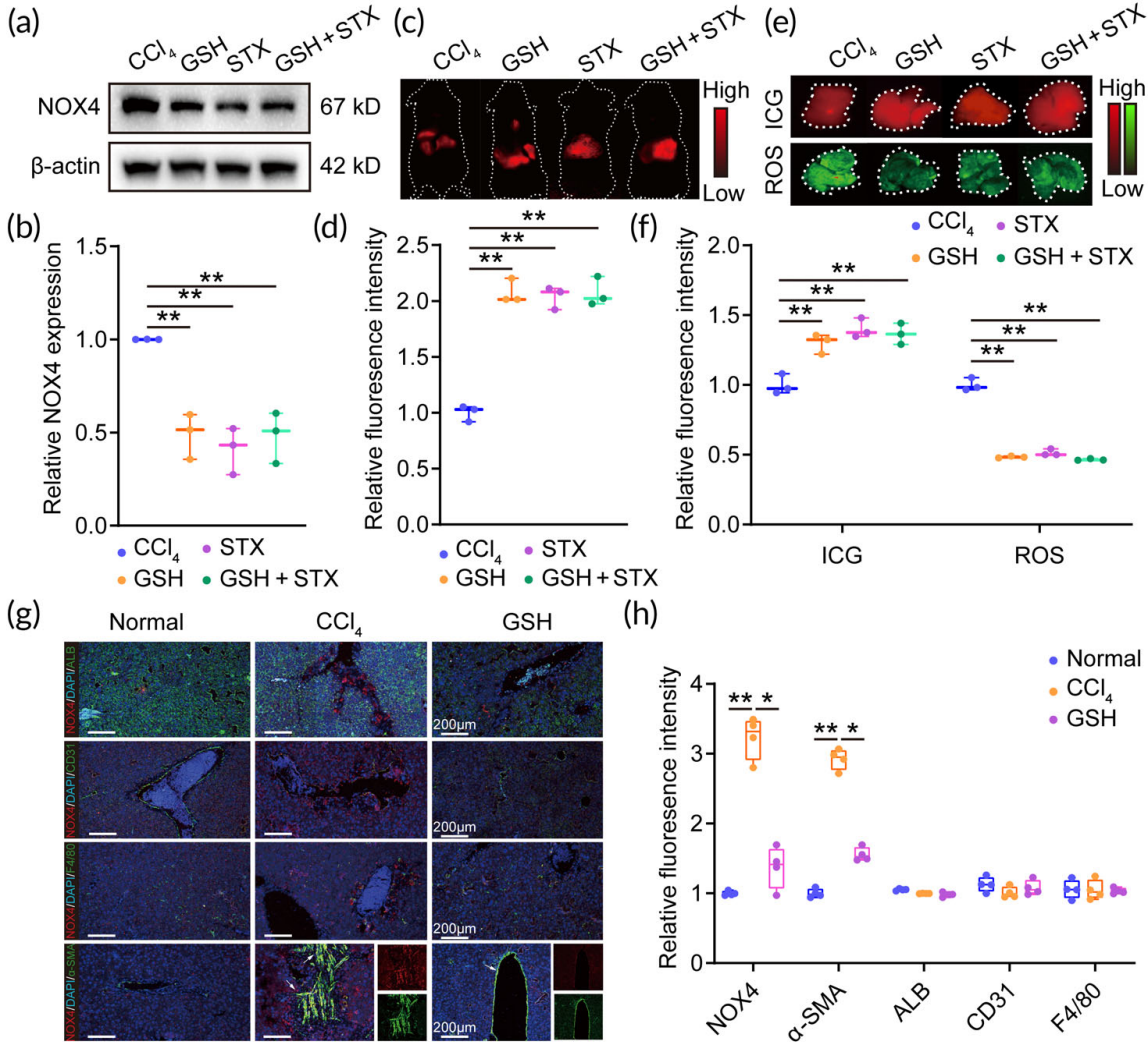
Glutathione (GSH) enhances adipose tissue‐derived mesenchymal stem cell (ADSC) engraftment efficiency by down regulating NOX4 expression. (a) Western blotting of NOX4 expression in liver tissues. (b) The relative NOX4 expression in liver tissues. (c) In vivo NIR imaging after ADSC transplantation for 3 days. (d) Relative fluorescence intensity of indocyanine green (ICG) signal in vivo. (e) Ex vivo NIR imaging and reactive oxygen species (ROS) content in liver tissues after cell transplantation for 3 days. (f) Relative fluorescence intensity of ICG signal and ROS in liver tissues. (g) Representative confocal laser scanning microscopy images of albumin (ALB), CD31, F4/80, and α‐SMA co‐located with NOX4 in liver tissues. Scale bars = 200 μm. (h) Quantification of NOX4, α‐SMA, ALB, CD31, and F4/80 expression in liver tissues. CCl_4_, carbon tetrachloride; STX, setanaxib. **, indicating *p* < 0.01; ***, indicating *p* < 0.001.

Given that NOXs are widely recognized as the major source of ROS generation,^34^ we also analyzed the content of NOXs in liver tissues. Strikingly, GSH treatment led to a significant decrease in the content of NOXs compared to the CCl_4_ treatment (Figure 2f). We next focused on NOX1, NOX2, and NOX4, which are the predominant homologs of NOXs in liver tissues,^35^ and we found that GSH exhibited a selective inhibition of NOX4 expression in liver tissues (Figure 2g,h). Meanwhile, we also found that GSH could down‐regulate NOX4 mRNA level in liver tissues (Figure S1), suggesting that the reduced NOX4 abundance of by GSH might be attributed to the down‐regulation at the transcriptional level. In order to further elucidate the regulatory effect of GSH on NOX4, we next utilized setanaxib (STX), a NOX4 inhibitor, to evaluate the ADSC engraftment efficiency and ROS content in vivo. Previously, it has been proven that STX could reduce NOX4 protein level.^36^, ^37^ Consistent with these results, the hepatic NOX4 could be effectively inhibited by the repeat STX treatment (for 14 days) in the current study, and the NOX4 inhibition could not be further enhanced by the combined treatment of GSH and STX (*n* = 3) (Figure 3a,b), indicating that there was no synergistic effect with the combined treatment of STX and GSH. Additionally, there were no significant differences in the ICG signal and ROS content among the GSH, STX, and combination groups (Figure 3c–f), suggesting that the enhanced cell efficiency of GSH is attributed to the down‐regulation of NOX4 expression.

In light of the NOX4 inhibition, we next evaluated the source of NOX4 in targeted cell types of liver tissues by co‐localization method. The immunofluorescence results show that the enhanced NOX4 expression is co‐localized in the α‐SMA‐positive cells (the activated HSCs), but not in ALB (a marker for hepatocytes), F4/80 (a marker for Kupffer cells), or CD31 (a marker for liver sinusoidal endothelial cells) positive cells; importantly, GSH effectively reduced NOX4 expression in α‐SMA‐positive cells (Figure 3g). Additionally, we also found that GSH reduced NOX4 and α‐SMA expression in liver tissues (Figure 3h). To further confirm the effect of GSH on NOX4 in HSCs, we also developed a reverse functional experiment in the activated HSCs (LX‐2 cells). As expected, GSH could effectively reduce NOX4 expression in activated HSCs; significantly, GSH could also down‐regulate the NOX4 expression in TGF‐β1‐treated HSCs (Figure S2). Taken together, these results suggest that the GSH down‐regulates NOX4 expression in activated HSCs.

### 2.4 GSH down‐regulates TGFβ1/SMAD3/NOX4 signaling pathway

**FIGURE 4:**
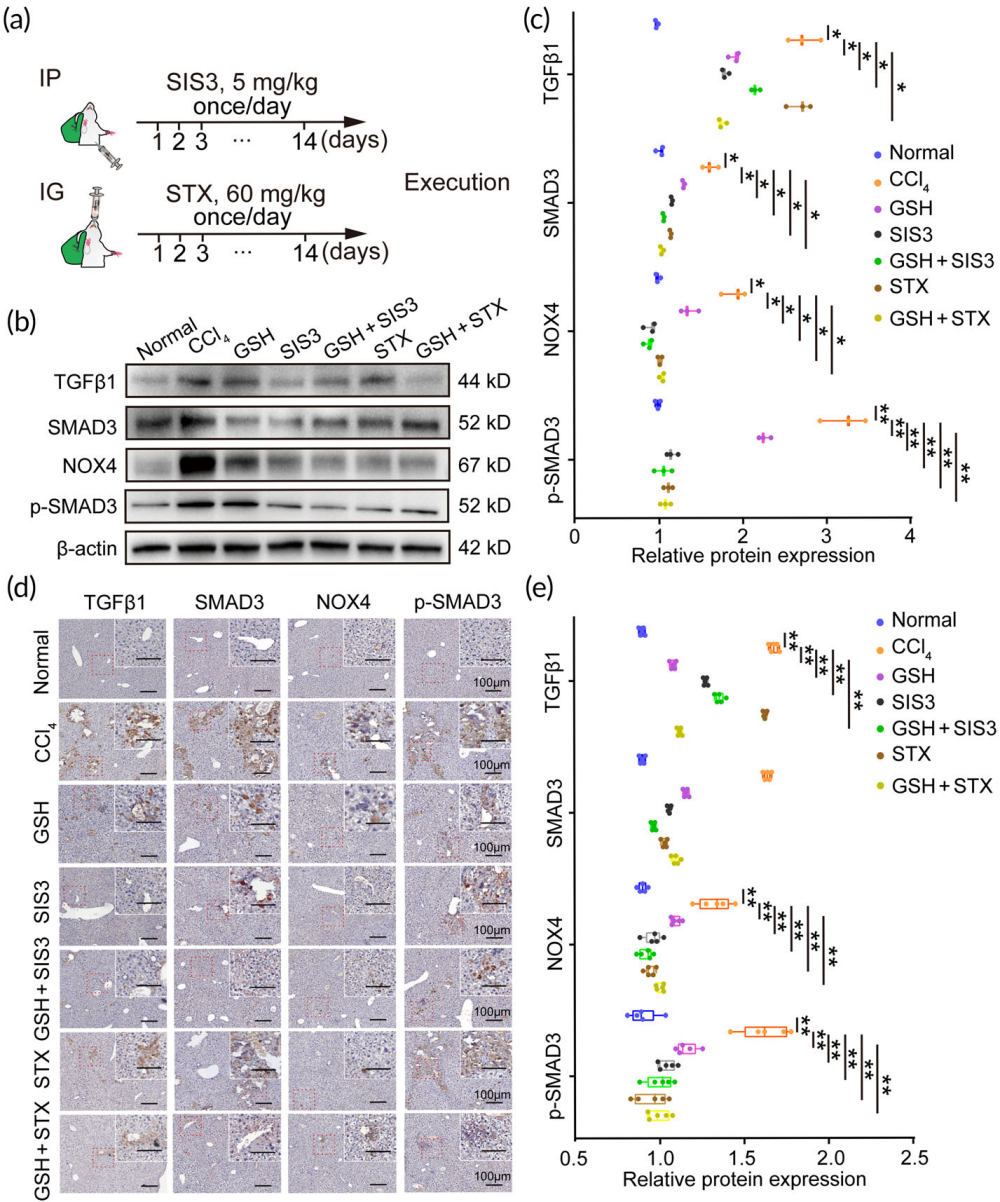
Glutathione (GSH) down‐regulates TGFβ1/SMAD3/NOX4 signaling in liver tissues. (a) Schedule of SIS3 and setanaxib (STX) administration in fibrotic mice (IP, intraperitoneal injection; IG, intragastric administration). (b) Western blotting of TGFβ1, SMAD3, p‐SMAD3, and NOX4 in liver tissues. (c) The relative TGFβ1, SMAD3, p‐SMAD3, and NOX4 expression. (d) Representative images of TGFβ1, SMAD3, p‐SMAD3, and NOX4 expression in liver tissues. Scale bars = 100 μm. (e) The relative TGFβ1, SMAD3, p‐SMAD3, and NOX4 expression. CCl_4_, carbon tetrachloride. *, indicating *p* < 0.05; **, indicating *p* < 0.01.

Based on the specific NOX4 inhibition of GSH, we further investigated the detailed mechanism by which GSH reduces NOX4 expression. It is widely acknowledged that the TGFβ1/SMAD3 pathway plays a key role in excessive ROS generation in liver fibrosis,^38^, ^39^ and TGFβ1 can regulate NOX4/ROS pathway through SMAD3 signaling.^32^ We therefore investigated the regulation of GSH on TGFβ1/SMAD3 signaling. Our findings showed that GSH reduces TGFβ1, SMAD3, and p‐SMAD3 in liver tissues as compared to the CCl_4_ treatment (Figure S3), suggesting that GSH down‐regulates TGFβ1/SMAD3 signaling. We next used SIS3 (a TGFβ1/SMAD3 pathway inhibitor), STX, and the combination with GSH to elucidate whether GSH reduces NOX4 expression through the down‐regulation of TGFβ1/SMAD3 signaling (Figure 4a). Significantly, we found that inhibition of TGFβ1/SMAD3 pathway resulted in down‐regulation of NOX4 expression (*n* = 5) (Figure 4b–e), implying that TGFβ1/SMAD3 pathway acts as an upstream regulator of NOX4. We also found the same inhibitory extent on NOX4 expression among GSH, SIS3, STX, and the combination (Figure 4b–e), suggesting that the reduced NOX4 expression by GSH is due to the down‐regulation of TGFβ1/SMAD3 signaling. Therefore, GSH down‐regulates TGFβ1/SMAD3/NOX4 signaling in liver tissues.

### 2.5 GSH improves ADSC engraftment efficiency by targeting TGFβ1/SMAD3/NOX4 signaling

**FIGURE 5:**
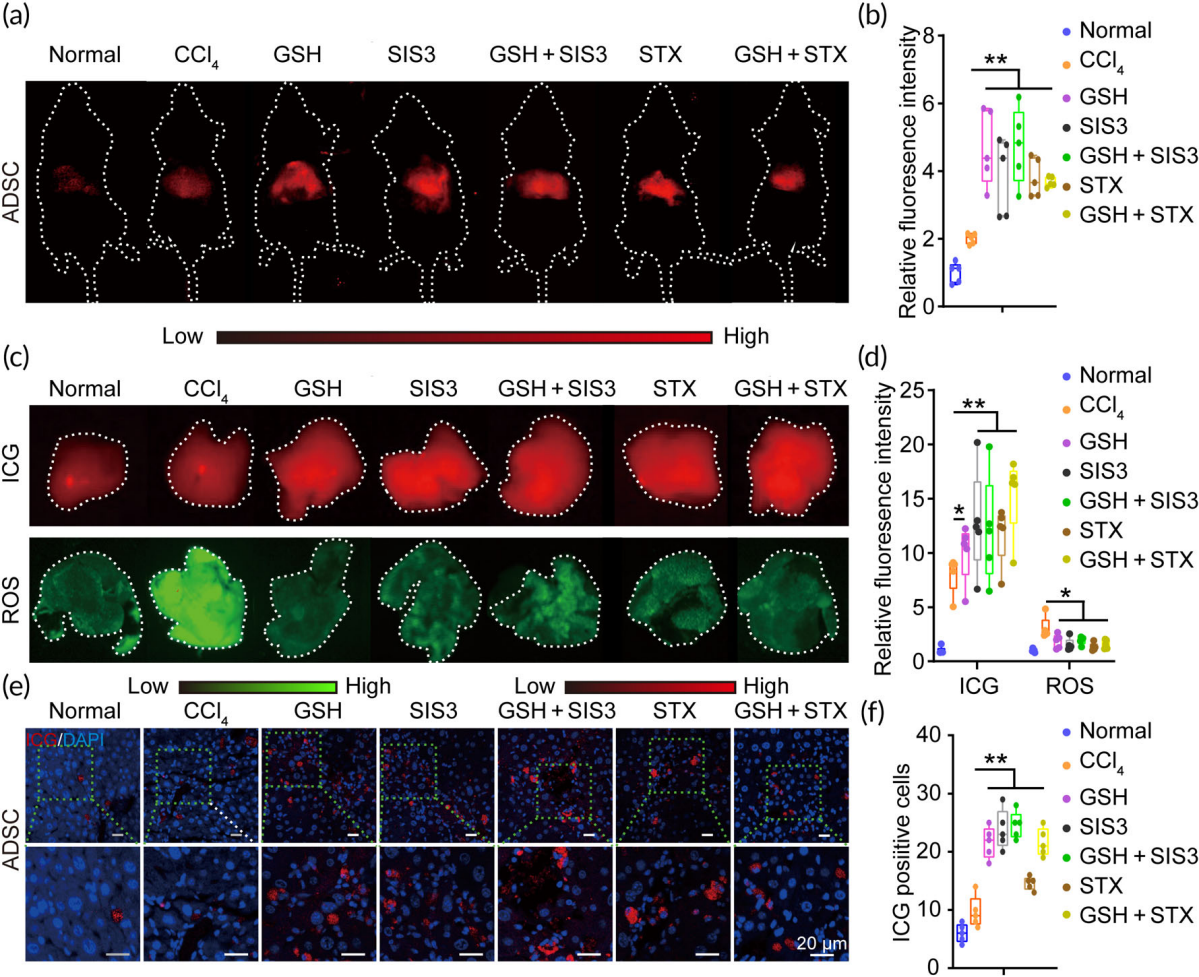
Glutathione (GSH) enhances adipose tissue‐derived mesenchymal stem cell (ADSC) engraftment efficiency through inhibiting TGFβ1/SMAD3/NOX4 pathway. (a) In vivo NIR imaging after ADSC transplantation for 3 days. (b) The relative fluorescence intensity of ADSCs in liver tissues after cell transplantation for 3 days. (c) Ex vivo NIR imaging and reactive oxygen species (ROS) content in liver tissues after cell transplantation for 3 days. (d) The relative fluorescence intensity of indocyanine green (ICG) signal and ROS in liver tissues after cell transplantation for 3 days. (e) Representative confocal laser scanning microscopy (CLSM) images of liver sections after ADSC transplantation for 3 days. Scale bars = 20 μm. (f) The ICG‐positive ADSCs after cell transplantation for 3 days. CCl_4_, carbon tetrachloride; STX, setanaxib. *, indicating *p* < 0.05; **, indicating *p* < 0.01.

We further investigated whether GSH improves ADSC engraftment efficiency and is closely related to TGFβ1/SMAD3/NOX4 signaling. In vivo NIR imaging showed a significantly increased ICG signal either by GSH, SIS3, STX, or the combination, and the relative ICG signal was no significant difference among these groups (*n* = 5) (Figure 5a,b). Meanwhile, these trends of signal coincided well with ex vivo NIR imaging and immunohistochemical analysis in liver tissues (Figure 5c–f). Additionally, we also found that GSH, SIS3, STX, and the combination inhibit hepatic ROS content as compared to CCl_4_ treatment. (Figure 5c,d). All these data suggest that inhibition of TGFβ1/SMAD3/NOX4 signaling benefits to improve ADSC engraftment efficiency. Together with the above results (Figures 3 and 4), it can be inferred that GSH could improve ADSC engraftment efficiency by targeting TGFβ1/SMAD3/NOX4 signaling pathway.

### 2.6 GSH enhances the therapeutic outcomes of ADSC therapy for liver fibrosis

**FIGURE 6:**
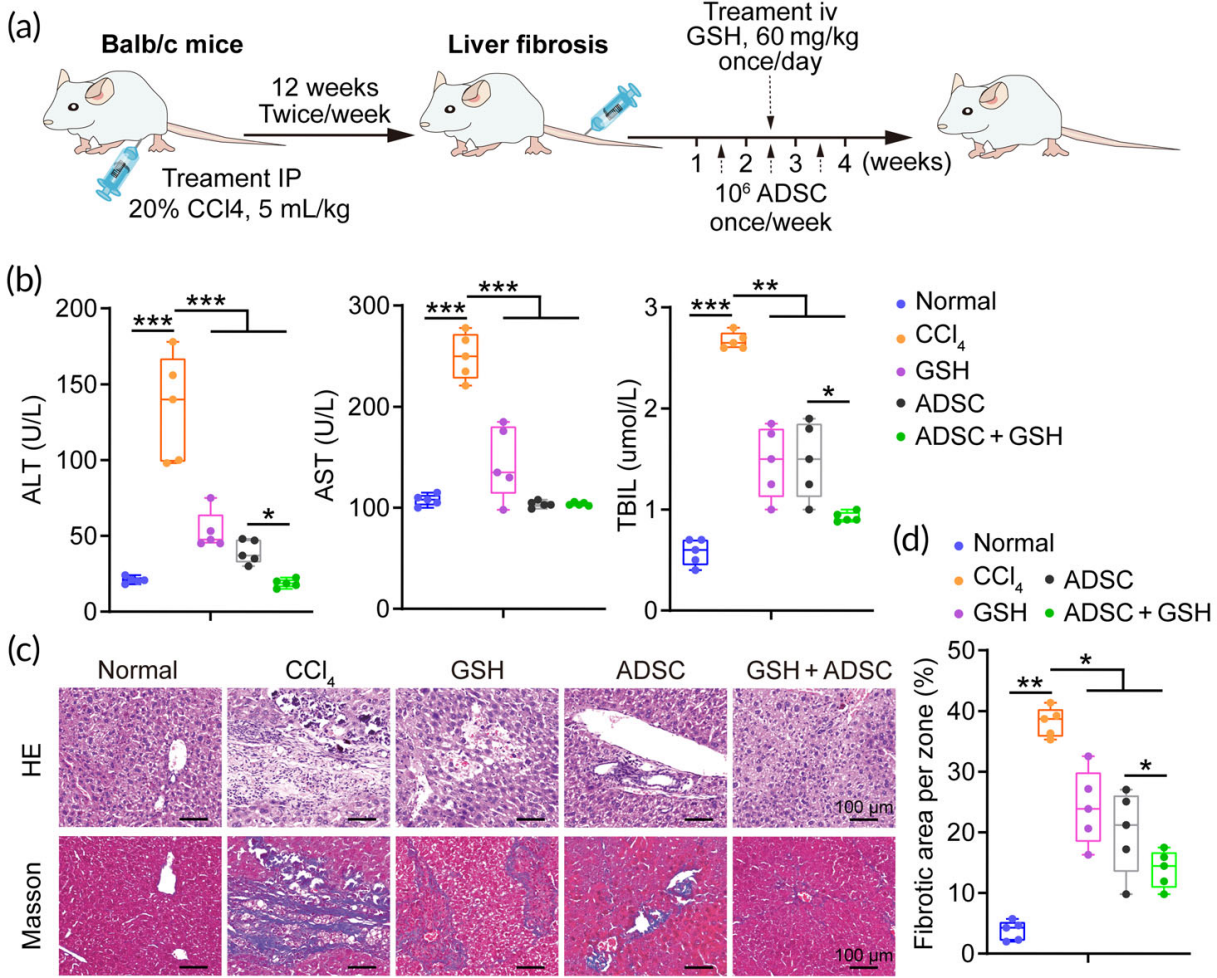
Glutathione (GSH) enhances the therapeutic outcomes of adipose tissue‐derived mesenchymal stem cell (ADSC) therapy for liver fibrosis. (a) Schedule of the ADSC therapy in a murine model of liver fibrosis. (b) The serum levels of ALT, AST, and TBIL after ADSC therapy. (c) Histopathological examination by hematoxylin and eosin (HE) and Masson staining, respectively. Scale bars = 100 μm. (d) The quantification of fibrotic area in liver tissues. CCl_4_, carbon tetrachloride; IP, intraperitoneal injection. *, indicating *p* < 0.05; **, indicating *p* < 0.01; ***, indicating *p* < 0.001.

Given the improved cell engraftment efficiency of GSH for ADSC transplantation in liver fibrosis, we next investigated whether GSH could enhance the therapeutic outcomes of ADSC therapy for liver fibrosis (Figure 6a). Comparing with the CCl_4_ treatment, the serum levels of aspartate aminotransferase (AST), alanine aminotransferase (ALT), and total bilirubin (TBIL) were significantly reduced by GSH treatment or ADSC therapy; importantly, the serum levels of ALT and TBIL could be further reduced by the combined treatment of GSH and ADSCs (*n* = 5) (Figure 6b), which means GSH enhances liver function recovery after ADSC therapy for liver fibrosis. In addition, the liver fibrotic degree was also markedly decreased in the combined groups as compared to those in the ADSC group (Figure 6c,d). In particular, the fibrotic area could be further reduced by the combined treatment of GSH and ADSCs compared with the ADSC group (Figure 6d). Taken together, these data suggested that GSH could enhance the therapeutic outcomes of ADSC therapy for liver fibrosis.

## 3 MATERIALS AND METHODS

### 3.1 Animals

Male Balb/c mice with specific pathogen free (SPF) status (aged 4–5 weeks; 18–20 g) were obtained from the Shanghai Slack Laboratory Animal Center. All animals were housed in SPF‐grade laboratory barrier facilities at 22–25°C and 55%–60% relative humidity and were handled in accordance with the guidelines specified in the Guide for the Care and Use of Laboratory Animals. The Institutional Animal Care and Use Committee of Mengchao Hepatobiliary Hospital of Fujian Medical University approved all animal experiments (NO. MHCC‐AEC‐2021‐01).

### 3.2 ADSC isolation and culture

The inguinal adipose tissues were obtained from the subcutaneous area of male Balb/c mice (aged 4–5 weeks) and cut into approximately 1 mm^3^ pieces. These tissue fragments were then digested with 0.1% type I collagenase (Sigma‐Aldrich, USA) in calcium‐containing HBSS (Hyclone, USA) for 1 h at 37°C. After digestion, the solution was filtered through a 100 μm cell strainer and neutralized with alpha minimal essential medium (α‐MEM) (Hyclone, USA) supplemented with 20% fetal bovine serum (FBS) (Gibco, USA). Osmotic lysates (Biyuntian Biological Co., Ltd., Shanghai, China) were utilized to remove red blood cells. Upon reaching 90% confluence, the cells were detached using 0.25% trypsin–ethylene diamine tetraacetic acid (EDTA) (Gibco, USA), and subsequently passaged at a 1:3 ratio. For the current study, passage 3 of ADSCs was used.

### 3.3 Cell labeling

For ADSC labeling, ADSCs were incubated with 1 μM CellTracker™ Cm‐dil (Qcbio Science&‐Technologies Co., Ltd., Shanghai, China) or ICG for 24 h. Following incubation, the ADSCs were washed with phosphate belanced solution (PBS) three times prior to intravenous transplantation.

### 3.4 Cell transplantation and tracking

To establish the liver fibrotic mouse model, CCl_4_ diluted in olive oil at a concentration of 20% was intraperitoneally injected into the mice at a dose of 5 mL/kg, twice a week for 12 weeks. Once fibrosis was established, the mice were randomly (random table method) divided into model (CCl_4_) and GSH groups. To track ADSC engraftment efficiency, all mice were treated with a tail vein injection of 1 × 10^6^ ICG‐labeled ADSCs per mouse. In vivo cell tracking was conducted using a NIR imaging system equipped with an 808 nm laser and an exposure time of 600 ms. To confirm the distribution of ADSCs, they were labeled with Cm‐dil and transplanted into fibrotic mice via tail vein injection at a dose of 10^6^ ADSCs per mouse. Following transplantation, all rats were euthanized with 100 mg/kg pentobarbital sodium, and the major organs, including the heart, liver, spleen, lungs, and kidneys, were collected for ADSC tracking using an inter‐vehicular information system (IVIS) (Bio‐Rad, USA). Moreover, liver tissues were sectioned at 10 μm and stained with 4’,6‐Diamidino‐2’‐phenylindole (DAPI) for further examination of cell engraftment using confocal laser scanning microscopy (LSM 780, Zeiss, Germany).

### 3.5 Drug treatment

The liver fibrotic mice were treated with a daily intravenous injection of 60 mg/kg GSH for a period of 4 weeks. Simultaneously, the mice underwent ADSC transplantation at a rate of 10^6^ ADSCs per mouse, once per week for a total of three treatments. After the completion of the combined treatment (3 days following the last ADSC transplantation), all rats were euthanized with 100 mg/kg pentobarbital sodium, and liver tissues were collected for histopathological examination, and sera were collected for biochemical analysis.

### 3.6 The combined therapy of GSH and ADSC transplantation for liver fibrosis

To enhance the engraftment efficiency of ADSCs, the liver fibrotic mice were first given a tail vein injection of 60 mg/kg GSH for 60 min prior to the transplantation of ADSCs. Subsequently, they were administered daily injections of GSH at the same dose for a period of 7 days. To investigate the potential mechanism underlying the enhanced engraftment, the fibrotic mice were treated with either SIS3, an inhibitor of TGFβ1/SMAD3 pathway, via intraperitoneal injection at a dosage of 5 mg/kg, or STX, an inhibitor of NOX4, through intragastric administration at a dosage of 60 mg/kg. Both treatments were given once daily for a duration of 2 weeks.

### 3.7 Histopathological assessment

The liver tissues were fixed in 4% paraformaldehyde at room temperature (RT) for 24 h. Following fixation, the tissues were dehydrated using ethanol and embedded in paraffin. The embedded tissues were then sliced into sections with a thickness of 5 μm. These sections were subsequently stained with hematoxylin and eosin (HE) or Masson's trichrome for histological examination. The examination was conducted using an ortho‐microscope from Zeiss, Germany.

### 3.8 Liver function assay

Orbital venous blood samples were collected, and the serum was separated by centrifugation at 1000 × *g* for 10 min at 4°C. The levels of ALT, AST, and TBIL in the serum were analyzed using commercial kits obtained from Nanjing Jiancheng Bioengineering Institute in Nanjing, China. The analysis was performed according to the manufacturer's protocol.

### 3.9 ROS content assay

Liver fibrotic mice were initially anesthetized by intraperitoneal injection of pentobarbital sodium at a dosage of 50 mL/kg (1 mg/mL). Subsequently, 100 μL of 25 mM DCFH‐DA was injected into the liver tissues through the hepatic portal vein and allowed to incubate for 30 min. The liver tissues were then collected, and the level of ROS was analyzed using an IVIS system (Bio‐Rad, USA) with an excitation wavelength of 488 nm.

### 3.10 Superoxide and NOXs content assay

The liver tissues were homogenized in normal saline at 4°C. The total protein content in the homogenized liver tissues was quantified using a bicinchoninic acid (BCA) assay kit (TransGen Biotech, Beijing, China). Subsequently, the levels of superoxide and NOXs in the liver tissues were evaluated using commercial kits (Nanjing Jiancheng Bioengineering Research Institute, Nanjing, China) following the manufacturer's protocol. Wst‐1 and wst‐8 assays were respectively used to detect the content of superoxide and NOXs.

### 3.11 Western blot analysis

The tissues were lysed in ice‐cold radio‐immunoprecipitation assay (RIPA) buffer (containing 0.5 M Tris–HCl, 10 mM EDTA, 1.5 M NaCl, 10% NP‐40, and 2.5% deoxycholic acid; pH, 7.4) containing the protease inhibitor phenylmethanesulfonyl fluoride (PMSF) (Beyotime Institute of Biotechnology, Shanghai, China). After quantifying the protein content using a BCA assay kit (TransGen Biotech, Beijing, China), equal amounts of protein (20 μg) were loaded onto a 10% sodium dodecyl sulfate polyacrylamide gel electrophoresis (SDS‐PAGE) gel for separation. Subsequently, the proteins in the gel were transferred onto nitrocellulose membranes (PALL, USA) using a transfer buffer (12 mM Tris base, 96 mM glycine, pH 8.3, and 20% methanol). The membranes were then blocked in rapid blocking buffer (TBST) containing 5% BSA for 2 h. Following the blocking step, the membranes were incubated separately with the antibodies for TGFβ1 (1:1000 dilution), SMAD3 (1:3000 dilution), p‐SMAD3 (1:2000 dilution), and NOX4 (1:4000 dilution), all of which were obtained from Proteintech (Wuhan, China), and β‐actin antibody (1:20,000 dilution; TransGen, Beijing, China) overnight at 4°C. After washing the membranes three times with TBST buffer, they were incubated with a horseradish peroxidase (HRP)‐conjugated secondary antibody (1:10,000 dilution; Santa Cruz Biotechnology) at RT for 1 h. Finally, the expression levels were analyzed by enhanced chemiluminescence and visualized using autoradiography.

### 3.12 Immunohistochemistry

After preliminary treatment of tissue section, the sections were drenched in citrate antigen retrieval solution (Beyotime Institute of Biotechnology, Shanghai, China) and heat‐treated in a pressure cooker for 2 min, natural cooling to RT, and washing with PBS buffer for three times. After that, the sections were performed within 3% H_2_O_2_ for 10 min and then were blocked in the PBS buffer containing 5% BSA for 30 min at RT. Next, the tissue sections were co‐incubated with the antibody of TGFβ1 (1:3000 dilution), SMAD3 (1:3000 dilution), p‐SMAD3 (1:2000 dilution), NOX4 (1:3000 dilution), CD31 (1:500 dilution), F4/80 (1:800 dilution), ALB (1:100 dilution), and alpha‐smooth muscle actin (α‐SMA) (1:1500 dilution), overnight at 4°C, respectively. Afterwards, the sections were washed three times with PBS buffer and incubated with secondary antibody at RT for 2 h. After three‐time washing with PBS, the samples were observed using an ortho‐microscope from Zeiss, Germany.

### 3.13 Quantification and statistical analyses

The data were presented as the mean ± standard deviation (SD). All statistical analyses were performed using GraphPad Prism version 9.0 (GraphPad Software, CA, USA). A Student's *t*‐test for unpaired samples was used to assess the statistical significance between two groups. For the analysis among multiple groups, a one‐way analysis of variance (ANOVA) with a suitable post hoc test was used. *p* < 0.05 was considered statistically significant.

## 4 CONCLUSIONS

Enhancing the efficiency of cell engraftment is crucial for the successful clinical translation of ADSC‐based therapies. Although various methods have been used to increase ADSC therapy for liver diseases,^40^, ^41^ there is still a lack of clinically potential strategies to enhance ADSC therapeutic efficacy. Oxidative stress is not only a characteristic feature of liver diseases but also a major factor contributing to the low survival rate of transplanted ADSCs.^42^ Therefore, using antioxidants could potentially increase transplanted cell survival rate. Endogenous GSH, which is highly expressed in liver tissues, is deficient during liver diseases, and exogenous administration of GSH is clinically used for hepatic protection.^43^ Previous studies have shown that GSH pretreatment can increase the ADSC viability in liver fibrosis.^21^ Consistent with this finding, our study further demonstrates that GSH significantly enhances the engraftment efficiency of ADSCs both in vivo and ex vivo, as confirmed by NIR fluorescence imaging. Importantly, GSH administration can further improve the therapeutic efficacy of ADSC therapy in liver fibrosis. Therefore, GSH pretreatment provides a novel and promising strategy to enhance the cell efficiency and therapeutic efficacy of ADSC therapy in liver diseases.

The survival of ADSCs is prone to being impaired by oxidative stress both in vitro and in vivo.^6^, ^44^ In this study, we found that the enhanced engraftment efficiency after GSH pretreatment was accompanied by a reduction in hepatic ROS levels, suggesting that reducing ROS generation is a potential mechanism by which GSH enhances the targeted delivery of ADSCs into fibrotic liver tissues. Since NOXs are the major source of excessive ROS generation in liver diseases,^33^ we further evaluated the effect of GSH on hepatic NOXs content. As predicted, we found that GSH treatment significantly inhibited NOXs and selectively inhibited NOX4 expression in activated HSCs of fibrotic liver tissues. Considering that NOX4 and ROS generation in activated HSCs play an important role in liver fibrosis,^45^, ^46^ GSH improves ADSC engraftment efficiency in liver fibrotic mice partly through inhibiting the NOX4/ROS pathway.

TGFβ1 plays a crucial role in HSC activation and the progression of liver fibrosis through a SMAD‐dependent mechanism.^30^ Previous studies have shown that SMAD3 can bind to the promoter region of NOX4, promoting ROS production and inflammation,^32^, ^47^ indicating that TGFβ1 can regulate the NOX4/ROS pathway by SMAD3.^48^ In the current study, GSH treatment reduced the protein level of TGFβ1, SMAD3, p‐SMAD3, and NOX4. Furthermore, the expression of these proteins was not affected by the addition of a TGFβ1/SMAD3 signaling inhibitor, confirming that the reduced ROS generation is partly through the down‐regulation of the TGFβ1/SMAD3/NOX4 pathway. Therefore, inhibiting the TGFβ1/SMAD3/NOX4 pathway is an effective strategy to reduce ROS generation in liver diseases, and the correlative work deserves further study. However, GSH did not affect the ratio of p‐SMAD3/SMAD3 as compared to those in SIS3, STX, or the combined treatment groups (Figure S4), suggesting that GSH might have a different regulator mechanism on TGFβ1/SMAD3 axis, and further studies should shed light on this discrepancy.

**FIGURE 7:**
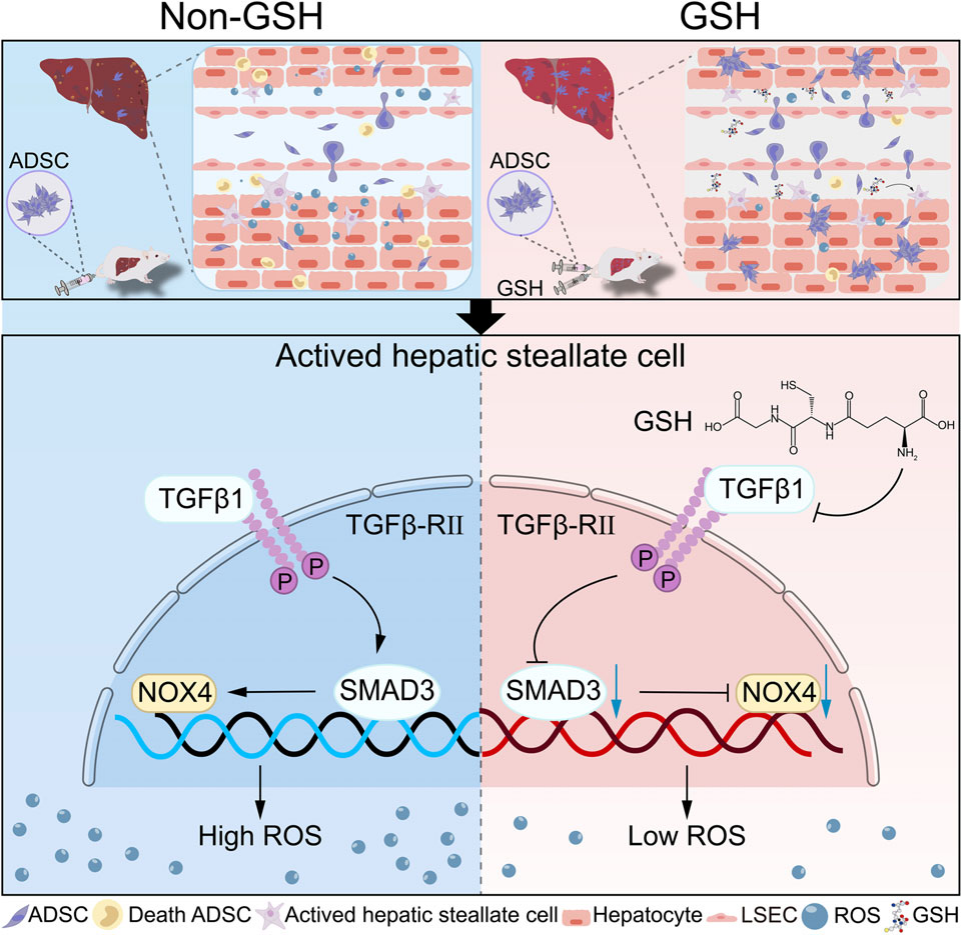
Schematic image illustrates the mechanism by which glutathione (GSH) improves adipose tissue‐derived mesenchymal stem cell (ADSC) engraftment efficiency in liver fibrosis. GSH improves ADSC engraftment efficiency by targeting the inhibiting TGFβ1/SMAD3/NOX4 pathway. ROS, reactive oxygen species; LSEC, liver sinusoidal endothelial cells.

Overall, our study demonstrates that GSH can improve the engraftment efficiency of ADSCs in liver fibrosis by inhibiting the TGFβ1/SMAD3/NOX4 signaling pathway (Figure 7). GSH pretreatment represents a novel potential clinical strategy to enhance ADSC transplantation.

## AUTHOR CONTRIBUTIONS

**Shaoxiong Yu**: data curation (lead); formal analysis (equal); writing—original draft (lead). **Yingchao Wang**: data curation (equal); writing—review and editing (equal). **Yingjun Shi**: Writing—review and editing (equal). **Saihua Yu**: Investigation (equal); writing—review and editing (equal). **Bixing Zhao**: Writing—review and editing (equal). **Naishun Liao**: Conceptualization (supporting); writing—review and editing (supporting). **Xiaolong Liu**: Conceptualization (supporting); writing—review and editing (supporting).

## CONFLICT OF INTEREST STATEMENT

The authors declare that they have no conflict of interest.

## Supporting information


**DATA S1.** Supporting Information.

## Data Availability

The data that support the findings of this study are available on request from the corresponding author.

## References

[1] Elzainy A , El Sadik A , Altowayan WM . Comparison between the regenerative and therapeutic impacts of bone marrow mesenchymal stem cells and adipose mesenchymal stem cells pre‐treated with melatonin on liver fibrosis. Biomolecules. 2024;14(3):297. doi:10.3390/biom14030297 38540717 PMC10968153

[2] Wen F , Yang G , Yu S , Liu H , Liao N , Liu Z . Mesenchymal stem cell therapy for liver transplantation: clinical progress and immunomodulatory properties. Stem Cell Res Ther. 2024;15(1):320. doi:10.1186/s13287-024-03943-6 39334441 PMC11438256

[3] Ji G , Zhang Z , Wang X , Guo Q , Zhang E , Li C . Comprehensive evaluation of the mechanism of human adipose mesenchymal stem cells ameliorating liver fibrosis by transcriptomics and metabolomics analysis. Sci Rep. 2024;14(1):20035. doi:10.1038/s41598-024-70281-1 39198546 PMC11358327

[4] Wang JL , Ding HR , Pan CY , Shi XL , Ren HZ . Mesenchymal stem cells ameliorate lipid metabolism through reducing mitochondrial damage of hepatocytes in the treatment of post‐hepatectomy liver failure. Cell Death Dis. 2021;12(1):111. doi:10.1038/s41419-020-03374-0 33479191 PMC7820227

[5] Zhang L , Ma XJ , Fei YY , et al. Stem cell therapy in liver regeneration: focus on mesenchymal stem cells and induced pluripotent stem cells. Pharmacol Ther. 2022;232:108004. doi:10.1016/j.pharmthera.2021.108004 34597754

[6] Liao N , Zhang D , Wu M , Yang H , Liu X , Song J . Enhancing therapeutic effects and in vivo tracking of adipose tissue‐derived mesenchymal stem cells for liver injury using bioorthogonal click chemistry. Nanoscale. 2020;13(3):1813‐1822. doi:10.1039/d0nr07272a 33433536

[7] Yu SX , Yu SH , Liu HY , Liao NS , Liu XL . Enhancing mesenchymal stem cell survival and homing capability to improve cell engraftment efficacy for liver diseases. Stem Cell Res Ther. 2023;14(1):235. doi:10.1186/s13287-023-03476-4 37667383 PMC10478247

[8] Yan W , Chen Y , Guo Y , et al. Irisin promotes cardiac homing of intravenously delivered MSCs and protects against ischemic heart injury. Adv Sci. 2022;9(7):e2103697. doi:10.1002/advs.202103697 PMC8895138 35038246

[9] Ni X , Ou C , Guo J , et al. Lentiviral vector‐mediated co‐overexpression of VEGF and Bcl‐2 improves mesenchymal stem cell survival and enhances paracrine effects in vitro. Int J Mol Med. 2017;40(2):418‐426. doi:10.3892/ijmm.2017.3019 28627637 PMC5505017

[10] Zheng J , Li H , He L , et al. Preconditioning of umbilical cord‐derived mesenchymal stem cells by rapamycin increases cell migration and ameliorates liver ischaemia/reperfusion injury in mice via the CXCR4/CXCL12 axis. Cell Prolif. 2019;52(2):e12546. doi:10.1111/cpr.12546 30537044 PMC6496237

[11] Xu YN , Xu W , Zhang X , et al. BM‐MSCs overexpressing the numb enhance the therapeutic effect on cholestatic liver fibrosis by inhibiting the ductular reaction. Stem Cell Res Ther. 2023;14(1):45. doi:10.1186/s13287-023-03276-w 36941658 PMC10029310

[12] Xu J , Liu X , Zhao F , Zhang Y , Wang Z . HIF1alpha overexpression enhances diabetic wound closure in high glucose and low oxygen conditions by promoting adipose‐derived stem cell paracrine function and survival. Stem Cell Res Ther. 2020;11(1):148. doi:10.1186/s13287-020-01654-2 32248837 PMC7132964

[13] Zhang X , Qin J , Wang X , et al. Netrin‐1 improves adipose‐derived stem cell proliferation, migration, and treatment effect in type 2 diabetic mice with sciatic denervation. Stem Cell Res Ther. 2018;9(1):285. doi:10.1186/s13287-018-1020-0 30359296 PMC6202825

[14] Song WJ , Liu PP , Zi Q , Shi JD , Hui XL . N‐acetylcysteine promotes the proliferation of porcine adipose derived stem cells during in vitro long‐term expansion for cultured meat production. Food Res Int. 2023;166:112606. doi:10.1016/j.foodres.2023.112606 36914351

[15] Ye Z , Lu W , Liang L , et al. Mesenchymal stem cells overexpressing hepatocyte nuclear factor‐4 alpha alleviate liver injury by modulating anti‐inflammatory functions in mice. Stem Cell Res Ther. 2019;10(1):149. doi:10.1186/s13287-019-1260-7 31133062 PMC6537220

[16] Prabha S , Merali C , Sehgal D , et al. Incorporation of paclitaxel in mesenchymal stem cells using nanoengineering upregulates antioxidant response, CXCR4 expression and enhances tumor homing. Mater Today Bio. 2023;19:100567. doi:10.1016/j.mtbio.2023.100567 PMC9898454 36747581

[17] Yang CS , Guo XS , Yue YY , Wang Y , Jin XL . Astaxanthin promotes the survival of adipose‐derived stem cells by alleviating oxidative stress via activating the nrf2 signaling pathway. Int J Mol Sci. 2023;24(4):3850. doi:10.3390/ijms24043850 36835263 PMC9959672

[18] Paik YH , Kim J , Aoyama T , de Minicis S , Bataller R , Brenner DA . Role of NADPH oxidases in liver fibrosis. Antioxid Redox Signal. 2014;20(17):2854‐2872. doi:10.1089/ars.2013.5619 24040957 PMC4026397

[19] Kantarcioglu M , Demirci H , Avcu F , et al. Efficacy of autologous mesenchymal stem cell transplantation in patients with liver cirrhosis. Turk J Gastroenterol. 2015;26(3):244‐250. doi:10.5152/tjg.2015.0074 26006200

[20] Liao N , Shi Y , Wang Y , et al. Antioxidant preconditioning improves therapeutic outcomes of adipose tissue‐derived mesenchymal stem cells through enhancing intrahepatic engraftment efficiency in a mouse liver fibrosis model. Stem Cell Res Ther. 2020;11(1):237. doi:10.1186/s13287-020-01763-y 32546282 PMC7298967

[21] Liao N , Su L , Cao Y , et al. Tracking cell viability for adipose‐derived mesenchymal stem cell‐based therapy by quantitative fluorescence imaging in the second near‐infrared window. ACS Nano. 2022;16(2):2889‐2900. doi:10.1021/acsnano.1c09960 35084178

[22] Schröder K . NADPH oxidases: current aspects and tools. Redox Biol. 2020;34:101512. doi:10.1016/j.redox.2020.101512 32480354 PMC7262010

[23] De Mochel N , Seronello S , Wang SH , et al. Hepatocyte NAD(P)H oxidases as an endogenous source of reactive oxygen species during hepatitis C virus infection. Hepatology. 2010;52(1):47‐59. doi:10.1002/hep.23671 20578128 PMC3141587

[24] Dubach VRA , San Segundo‐Acosta P , Murphy BJ . Structural and mechanistic insights into Streptococcus pneumoniae NADPH oxidase. Nat Struct Mol Biol. 2024;31:1769‐1777. doi:10.1038/s41594-024-01348-w 39039317 PMC11564096

[25] Penuelas‐Haro I , Espinosa‐Sotelo R , Crosas‐Molist E , et al. The NADPH oxidase NOX4 regulates redox and metabolic homeostasis preventing HCC progression. Hepatology. 2022;2(78):416‐433. doi:10.1002/hep.32702 PMC10344438 35920301

[26] Pecchillo Cimmino T , Ammendola R , Cattaneo F , Esposito G . NOX dependent ROS generation and cell metabolism. Int J Mol Sci. 2023;24(3):2086. doi:10.3390/ijms24032086 36768405 PMC9916913

[27] Cheng Q , Li C , Yang CF , et al. Methyl ferulic acid attenuates liver fibrosis and hepatic stellate cell activation through the TGF‐beta1/Smad and NOX4/ROS pathways. Chem Biol Interact. 2019;299:131‐139. doi:10.1016/j.cbi.2018.12.006 30543783

[28] Matuz‐Mares D , Vázquez‐Meza H , Vilchis‐Landeros MM . NOX as a therapeutic target in liver disease. Antioxidants. 2022;11(10):2038. doi:10.3390/antiox11102038 36290761 PMC9598239

[29] Avila MA , Lan T , Kisseleva T , Brenner DA . Deficiency of NOX1 or NOX4 prevents liver inflammation and fibrosis in mice through inhibition of hepatic stellate cell activation. PLoS One. 2015;10(7):e0129743. doi:10.1371/journal.pone.0129743 26222337 PMC4519306

[30] Richter K , Konzack A , Pihlajaniemi T , Heljasvaara R , Kietzmann T . Redox‐fibrosis: impact of TGFbeta1 on ROS generators, mediators and functional consequences. Redox Biol. 2015;6:344‐352. doi:10.1016/j.redox.2015.08.015 26335400 PMC4565043

[31] Hung CT , Su TH , Chen YT , et al. Targeting ER protein TXNDC5 in hepatic stellate cell mitigates liver fibrosis by repressing non‐canonical TGFβ signalling. Gut. 2022;71(9):1876‐1891. doi:10.1136/gutjnl-2021-325065 34933915

[32] Wang JN , Yang Q , Yang C , et al. Smad3 promotes AKI sensitivity in diabetic mice via interaction with p53 and induction of NOX4‐dependent ROS production. Redox Biol. 2020;32:101479. doi:10.1016/j.redox.2020.101479 32143149 PMC7058410

[33] Che ZD , Zhou Z , Li SQ , Gao L , Xiao J , Wong NK . ROS/RNS as molecular signatures of chronic liver diseases. Trends Mol Med. 2023;29(11):951‐967. doi:10.1016/j.molmed.2023.08.001 37704494

[34] Aviello G , Knaus UG . NADPH oxidases and ROS signaling in the gastrointestinal tract. Mucosal Immunol. 2018;11(4):1011‐1023. doi:10.1038/s41385-018-0021-8 29743611

[35] Mortezaee K . Nicotinamide adenine dinucleotide phosphate (NADPH) oxidase (NOX) and liver fibrosis: a review. Cell Biochem Funct. 2018;36(6):292‐302. doi:10.1002/cbf.3351 30028028

[36] Zheng H , Xu N , Zhang Z , Wang F , Xiao J , Ji X . Setanaxib (GKT137831) ameliorates doxorubicin‐induced cardiotoxicity by inhibiting the NOX1/NOX4/reactive oxygen species/MAPK pathway. Front Pharmacol. 2022;13:823975. doi:10.3389/fphar.2022.823975 35444554 PMC9014097

[37] Liao J , Lai Z , Huang G , et al. Setanaxib mitigates oxidative damage following retinal ischemia‐reperfusion via NOX1 and NOX4 inhibition in retinal ganglion cells. Biomed Pharmacother. 2024;170:116042. doi:10.1016/j.biopha.2023.116042 38118351

[38] Balah A , Ezzat O , Akool ES . Vitamin E inhibits cyclosporin A‐induced CTGF and TIMP‐1 expression by repressing ROS‐mediated activation of TGF‐β/Smad signaling pathway in rat liver. Int Immunopharmacol. 2018;65:493‐502. doi:10.1016/j.intimp.2018.09.033 30391882

[39] Ding SB , Yu L , An B , Zhang G , Yu P , Wang Z . Combination effects of airborne particulate matter exposure and high‐fat diet on hepatic fibrosis through regulating the ROS‐endoplasmic reticulum stress‐TGFβ/SMADs axis in mice. Chemosphere. 2018;199:538‐545. doi:10.1016/j.chemosphere.2018.02.082 29455124

[40] Liu N , Zhou ZY , Ning X , et al. Enhancing the paracrine effects of adipose stem cells using nanofiber‐based meshes prepared by light‐welding for accelerating wound healing. Mater Des. 2023;225:111582. doi:10.1016/j.matdes.2022.111582

[41] Yuan X , Li L , Liu H , et al. Strategies for improving adipose‐derived stem cells for tissue regeneration. Burns. Dent Traumatol. 2022;10:tkac028. doi:10.1093/burnst/tkac028 PMC9382096 35992369

[42] Jiang L , Chen HY , He CH , et al. Dual‐modal apoptosis assay enabling dynamic visualization of ATP and reactive oxygen species in living cells. Anal Chem. 2023;95(6):3507‐3515. doi:10.1021/acs.analchem.2c05671 36724388

[43] Vairetti M , Di Pasqua LG , Cagna M , Richelmi P , Ferrigno A , Berardo C . Changes in glutathione content in liver diseases: an update. Antioxidants. 2021;10(3):364. doi:10.3390/antiox10030364 33670839 PMC7997318

[44] Liao N , Shi Y , Zhang C , et al. Antioxidants inhibit cell senescence and preserve stemness of adipose tissue‐derived stem cells by reducing ROS generation during long‐term in vitro expansion. Stem Cell Res Ther. 2019;10(1):306. doi:10.1186/s13287-019-1404-9 31623678 PMC6798439

[45] Zhou M , Zhao X , Liao L , et al. Forsythiaside a regulates activation of hepatic stellate cells by inhibiting NOX4‐dependent ROS. Oxid Med Cell Longev. 2022;2022:9938392. doi:10.1155/2022/9938392 35035671 PMC8754607

[46] Du JJ , Sun JC , Li N , Li X‐Q , Sun W‐Y , Wei W . Beta‐Arrestin2 deficiency attenuates oxidative stress in mouse hepatic fibrosis through modulation of NOX4. Acta Pharmacol Sin. 2021;42(7):1090‐1100. doi:10.1038/s41401-020-00545-9 33116250 PMC8209231

[47] Boudreau HE , Casterline BW , Rada B , Korzeniowska A , Leto TL . Nox4 involvement in TGF‐beta and SMAD3‐driven induction of the epithelial‐to‐mesenchymal transition and migration of breast epithelial cells. Free Radic Biol Med. 2012;53(7):1489‐1499. doi:10.1016/j.freeradbiomed.2012.06.016 22728268 PMC3448829

[48] Li H , Ren J , Cui H , et al. Dexamethasone induces senescence‐associated changes in trabecular meshwork cells by increasing ROS levels via the TGFbeta/Smad3‐NOX4 Axis. Cell Transplant. 2023;32:9636897231177356. doi:10.1177/09636897231177356 37265069 PMC10272683

