# Supplementary material for: Reduced glutathione enhances adipose tissue‐derived mesenchymal stem cell engraftment efficiency for liver fibrosis by targeting TGFβ1/SMAD3/NOX4 pathway
Source: Bioeng Transl Med. 2024 Dec 10;10(2):e10735. doi: 10.1002/btm2.10735 (PMC11883125; doi:10.1002/btm2.10735)
Supplement: Supplementary file 1 — DATA S1. Supporting Information. [file BTM2-10-e10735-s001.docx]

**Reduced glutathione enhances adipose tissue-derived mesenchymal stem cell engraftment efficiency for liver fibrosis by targeting** **TGFβ1/SMAD3/NOX4 pathway**

**Shaoxiong Yu^1, 2, 3^, Yingchao Wang^1, 2, 3^, Yingjun Shi^1, 2, 3^, Saihua Yu^1, 2, 3^, Bixing Zhao^1, 2, 3^, Naishun Liao^1, 2, 3,*^, Xiaolong Liu^1, 2, 3,*^**

1. The United Innovation of Mengchao Hepatobiliary Technology Key Laboratory of Fujian Province, Mengchao Hepatobiliary Hospital of Fujian Medical University, Fuzhou 350028, China.

2. Mengchao Med-X Center, Fuzhou University, Fuzhou, 350116, China.

3. The Liver Center of Fujian Province, Fujian Medical University, Fuzhou 350028, China.

**Reverse functional assay *in vitro***

LX2 cells were purchased from Bogu Biotech Co., Ltd (Shanghai, China), and cultured in 6-well plates the complete medium containing RPMI 1640 and 10% FBS. The cells were treated with 10 ng/mL TGF-β1 and 10 μΜ GSH for 24 hours. After that, all cells were collected, and the total protein was isolated for Western blot assay.

**Quantitative real-time PCR analysis**

Total RNA was collected using a TRIzol reagent kit (TransGen Biotech, China) following the manufacturer's instructions. Afterwards, mRNA was reversely transcribed into cDNA using a cDNA synthesis kit (Roche, Germany). The quantitative real-time PCR analysis was performed in an ABI step one plus real-time PCR system (Carlsbad, USA), the PCR conditions were as follows: 95 °C for 15 sec, 60 °C for 30 sec, 70 °C for 30 sec, repeating 40 cycles. The primer sequences are as follows: NOX4 forward, 5’- CCCTCCTGGCTGCATTAGTC-3’; NOX4 reverse, 5’- ACCCCTCGAGGCAAAGATCC-3’. β-actin forward, 5’-CTGAGAGGGAAATCGTGCGT-3’; β-actin reverse, 5’-TGTTGGCATAGAGGTCTTTACGG-3. The 2^-△△Ct^ formula was used to analyze the relative gene expression.

**
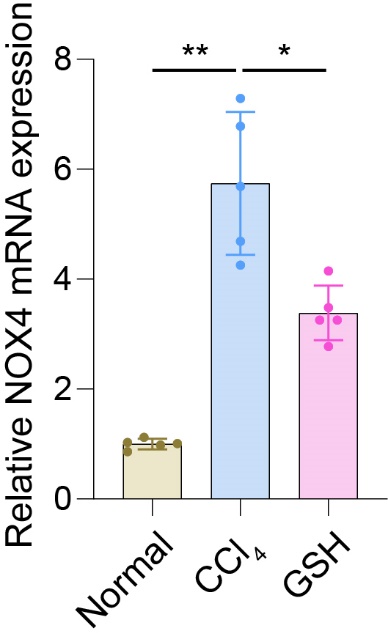
**

**SUPPLEMENTARY FIGURE 1** GSH down-regulates NOX4 mRNA expression in liver tissues.

**
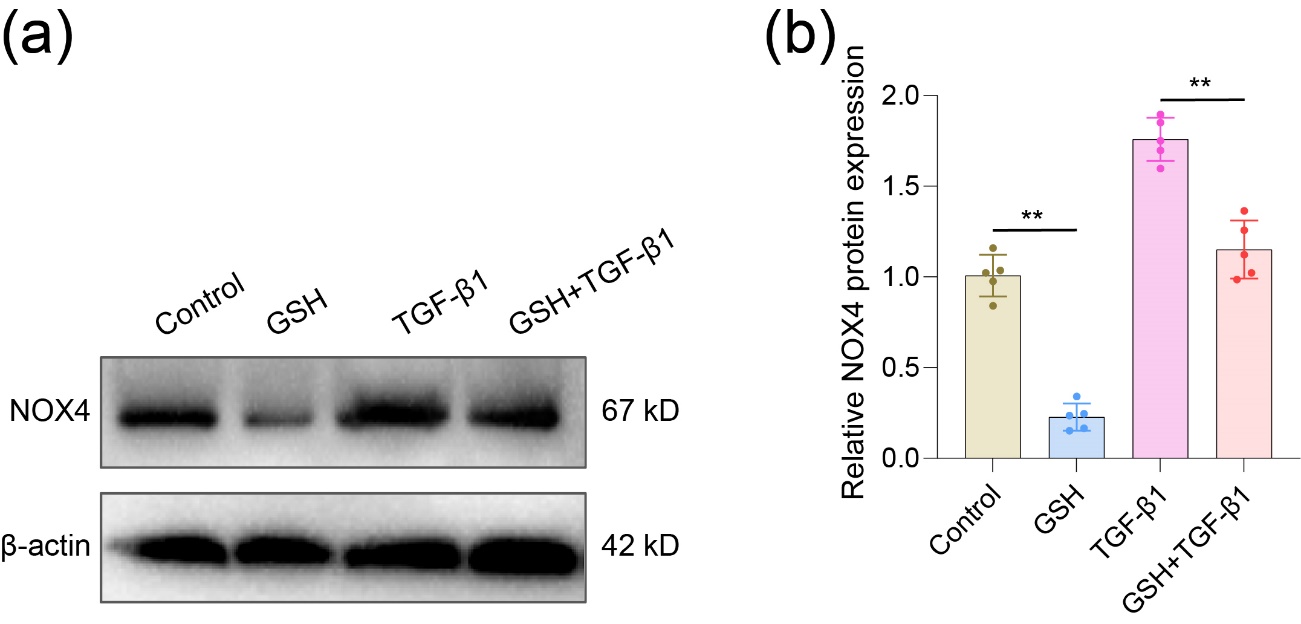
**

**SUPPLEMENTARY FIGURE 2** GSH down-regulates NOX4 expression in LX2 cells. (a) The NOX4 protein expression in LX2 or TGF-β1-treated LX2 cells. (b) The relative NOX4 protein expression.


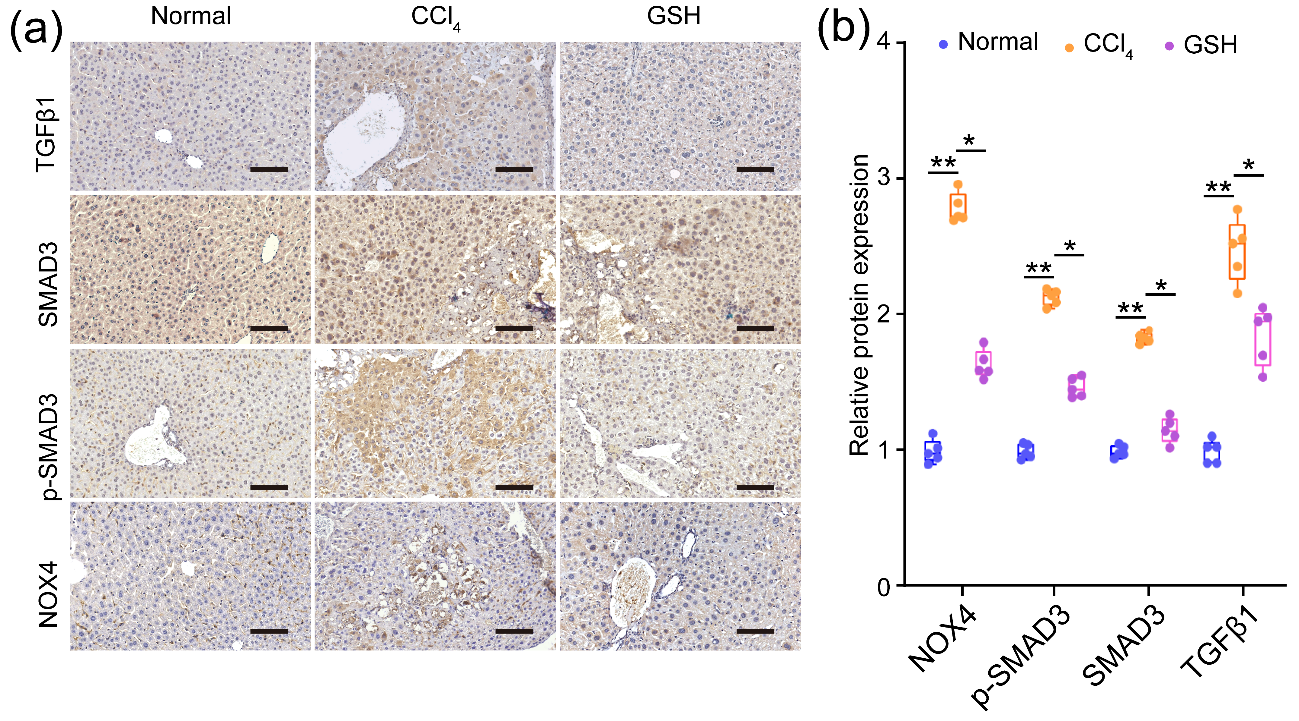


**SUPPLEMENTARY FIGURE 3** GSH down-regulates the TGFβ1/SMAD3 signaling. (a) Representative images of TGFβ1, SMAD3, p-SMAD3, and NOX4 expression in liver tissues. Scale bars = 100 μm. (b) The relative TGFβ1, SMAD3, p-SMAD3, and NOX4 expression.


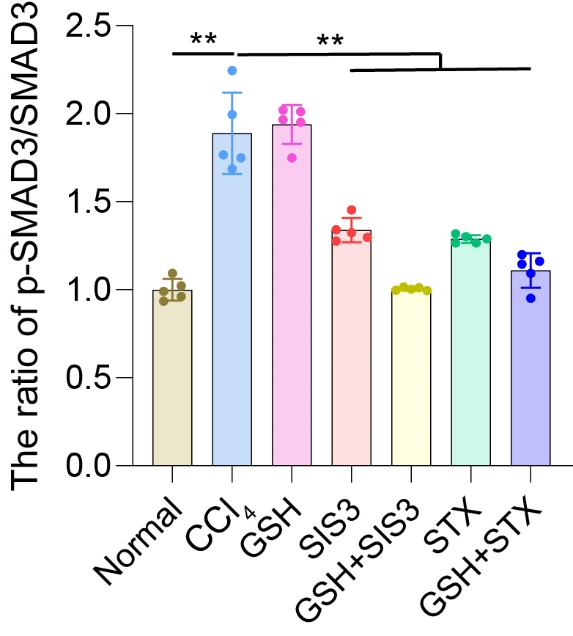


**SUPPLEMENTARY FIGURE 4** The ratio of p-SMAD3/SMAD3 after GSH treatment in liver tissues.
